# Retracted: *Scolopendra subspinipes mutilans L. Koch* Ameliorates Rheumatic Heart Disease by Affecting Relative Percentages of CD4^+^CD25^+^FoxP3 Treg and CD4^+^IL17 T Cells

**DOI:** 10.1155/2021/9816302

**Published:** 2021-11-03

**Authors:** Evidence-Based Complementary and Alternative Medicine

*Evidence-Based Complementary and Alternative Medicine* has retracted the article titled “*Scolopendra subspinipes mutilans L. Koch* Ameliorates Rheumatic Heart Disease by Affecting Relative Percentages of CD4^+^CD25^+^FoxP3 Treg and CD4^+^IL17T Cells” [1] at the request of the authors, due to the errors in data collection and analysis. The authors have identified that the ELISA kit used to measure the concentration of IL-6 expired in July 2016 causing this result to be unreliable. Additionally, the authors note that 100 mg of SSLK powder was administered to patients, while the recommended dose as per the Chinese Pharmacopoeia 2000 Edition is 3–5 g [2].

The authors apologised for this error, which they feel invalidates the conclusions and cannot be corrected. With the agreement of the editorial board and the authors, the article is retracted from the journal.

## References

[1] Jiang T., Zhao Q., Sun H. (2019). *Scolopendra subspinipes mutilans L. Koch* Ameliorates Rheumatic Heart Disease by Affecting Relative Percentages of CD4^+^CD25^+^FoxP3 Treg and CD4^+^IL17 T Cells. *Evidence-Based Complementary and Alternative Medicine*.

[2] National Pharmacopoeia Committee *Chinese Pharmacopoeia 2000 Edition, Page 294*.

